# Supplementary figures and images for: Phospholipase A2 activity is required for immune defense of European (Apis mellifera) and Asian (Apis cerana) honeybees against American foulbrood pathogen, Paenibacillus larvae
Source: PLoS One. 2024 Feb 6;19(2):e0290929. doi: 10.1371/journal.pone.0290929 (PMC10846722; doi:10.1371/journal.pone.0290929)

**(A)**


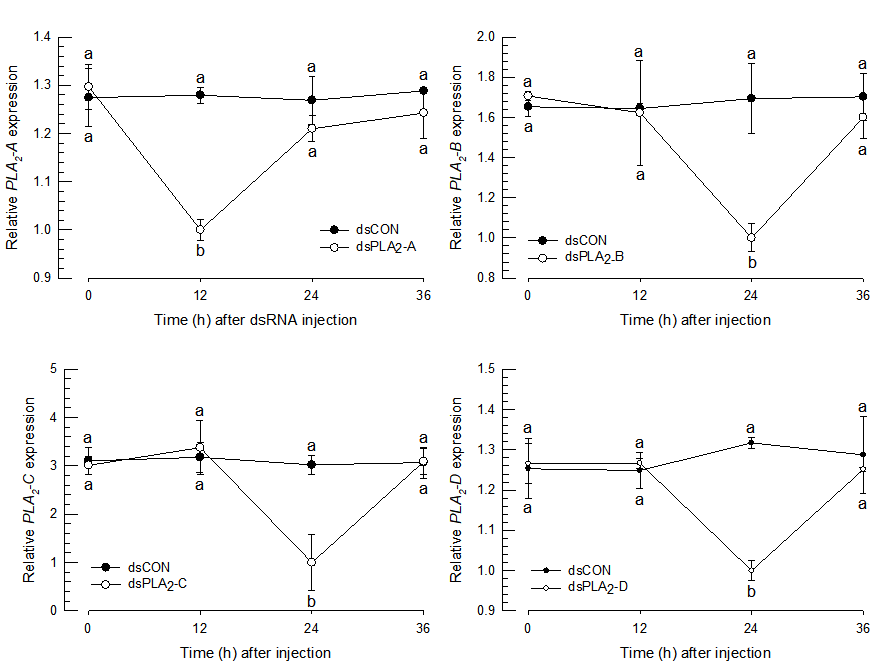


**(B)**


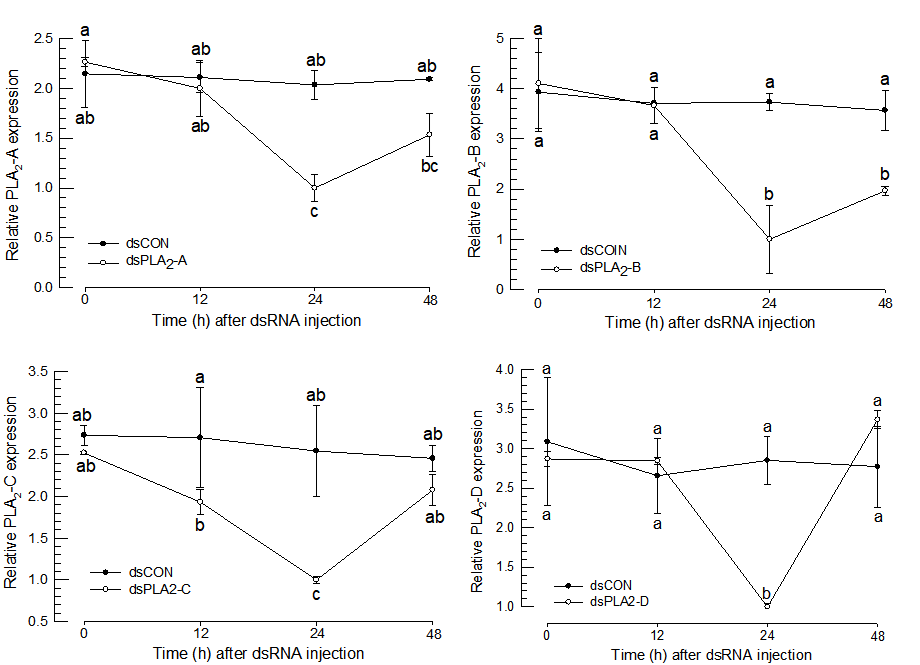

Supplement: S1 Fig — (A) Change of PLA2 expression levels in A. mellifera after injection (1 μg/larva) of dsRNA (‘dsPLA2-A’, ‘dsPLA2-B’, ‘dsPLA2-C’, ‘dsPLA2-D’). (B) Change of PLA2 expression levels in A. cerana after injection (1 μg/larva) of dsRNA (‘dsPLA2-A’, ‘dsPLA2-B’, ‘dsPLA2-C’, ‘dsPLA2-D’). GFP was used as a control dsRNA (‘dsCON’). (DOCX) [file pone.0290929.s001.docx]
