# Supplementary material for: Phospholipase A2 activity is required for immune defense of European (Apis mellifera) and Asian (Apis cerana) honeybees against American foulbrood pathogen, Paenibacillus larvae
Source: PLoS One. 2024 Feb 6;19(2):e0290929. doi: 10.1371/journal.pone.0290929 (PMC10846722; doi:10.1371/journal.pone.0290929)
Supplement: S1 Table — (DOCX) [file pone.0290929.s002.docx]

**Table S1**. List of primers used in this study

| Genes | Primers (5’→3’) | Annealing temperature (℃) | | Product size (bp) |
| --- | --- | --- | --- | --- |
| Am-PLA2A-QPCR-F1 | AGCTAGGTCGGTTCAAGCAC | 53 | | 480 |
| Am-PLA2A-QPCR-R1 | ACCCATTGGTACACGCTCTG |  |  |  |
| Am-PLA2B-QPCR-F1 | TTGGCTTATGCGTGGTCAGG | 53 | | 293 |
| Am-PLA2B-QPCR-R1 | CCACATCCGTTACTTTGCGG |  |  |  |
| Am-PLA2C-QPCR-F1 | TTTCCTTCTTTTCTTCCGATC | 53 | | 255 |
| Am-PLA2C-QPCR-R1 | GGAAGATCACCTCCAAGGGC |  |  |  |
| Am-PLA2D-QPCR-F1 | GGCGATGGCGGTAGTCAAA | 53 | | 344 |
| Am-PLA2D-QPCR-R1 | ACAAATGGATCACCCCAGCC |  |  |  |
| Ac-PLA2A-QPCR-F1 | AATTAAATGCGGATGCGCCC | 53 | | 413 |
| Ac-PLA2A-QPCR-R1 | GAACTTCGCATCGCAGTCAC |  |  |  |
| Ac-PLA2B-QPCR-F1 | TTGGCTTATGCGTGGTCAGG | 53 | | 293 |
| Ac-PLA2B-QPCR-R1 | CCACATCCGTTACTTTGCGG |  |  |  |
| Ac-PLA2C-QPCR-F1 | AGCGGAGATGTGGAAACAGG | 53 | 300 | |
| Ac-PLA2C-QPCR-R1 | TTGAACAAGTACCGGAACTTT |  |  |  |
| Ac-PLA2D-QPCR-F1 | GGCGATGGCGGTAGTCAAA | 53 | | 344 |
| Ac-PLA2D-QPCR-R1 | ACAAATGGATCACCCCAGCC |  |  |  |
| Am-T7-PLA2A-F1 | TAATACGACTCACTATAGGGAGAAGCTAGGTCGGTTCAAGCAC | 55 | | 512 |
| Am-T7- PLA2A-R1 | TAATACGACTCACTATAGGGAGAACCCATTGGTACACGCTCTG |  |  |  |
| Am-T7-PLA2B-F1 | TAATACGACTCACTATAGGGAGATTGGCTTATGCGTGGTCAGG | 55 | | 32 |
| Am-T7-PLA2B-R1 | TAATACGACTCACTATAGGGAGACCACATCCGTTACTTTGCGG |  |  |  |
| Am-T7-PLA2C-F1 | TAATACGACTCACTATAGGGAGAACGTGGACTCTGGCACATTT | 55 | | 287 |
| Am-T7-PLA2C-R1 | TAATACGACTCACTATAGGGAGAGGAAGATCACCTCCAAGGGC |  |  |  |
| Am-T7-PLA2D-F1 | TAATACGACTCACTATAGGGAGAGGCGATGGCGGTAGTCAAA | 55 | | 376 |
| Am-T7-PLA2D-R1 | TAATACGACTCACTATAGGGAGAACAAATGGATCACCCCAGCC |  |  |  |
| Ac-T7-PLA2A-F1 | TAATACGACTCACTATAGGGAGAAATTAAATGCGGATGCGCCC | 55 | | 445 |
| Ac-T7-PLA2A-R1 | TAATACGACTCACTATAGGGAGAGAACTTCGCATCGCAGTCAC |  |  |  |
| Ac-T7-PLA2B-F1 | TAATACGACTCACTATAGGGAGATTGGCTTATGCGTGGTCAGG | 55 | | 325 |
| Ac-T7-PLA2B-R1 | TAATACGACTCACTATAGGGAGACCACATCCGTTACTTTGCGG |  |  |  |
| Ac-T7-PLA2C-F1 | TAATACGACTCACTATAGGGAGAAGCGGAGATGTGGAAACAGG | 55 | | 332 |
| Ac-T7-PLA2C-R1 | TAATACGACTCACTATAGGGAGAATCGCCATAATGTGCAACGC |  |  |  |
| Ac-T7-PLA2D-F1 | TAATACGACTCACTATAGGGAGAGGCGATGGCGGTAGTCAAA | 55 | | 376 |
| Ac-T7-PLA2D-R1 | TAATACGACTCACTATAGGGAGAACAAATGGATCACCCCAGCC |  |  |  |
| RPI32-F | CGTCACCAGAGTGATCGTTACA | 53 | | 250 |
| RPI32-R- | CCCCATGAGCAATTTCAGCAC |  |  |  |
